# Smoking, alcohol consumption and risk of Dupuytren’s disease: a Mendelian randomization study

**DOI:** 10.1186/s12920-023-01650-4

**Published:** 2023-09-07

**Authors:** Zifeng Wang, Zhenyu Wang, Zijian Yan, Zhujie Xu, Aiguo Gao

**Affiliations:** 1 Department of Orthopedics, The Affiliated Wuxi People’s Hospital of Nanjing Medical University, Wuxi, 214000 China; 2 Department of Orthopedics, Taicang Shaxi People’s Hospital, Taicang, 215400 China; 3 The First Clinical Medical College, Guangzhou University of Chinese Medicine, Guangzhou, 510405 China

**Keywords:** Smoking, Alcohol consumption, Causal inference, Dupuytren's disease, Mendelian randomization, Risk factors

## Abstract

**Background:**

The correlation between smoking and alcohol consumption and the development of Dupuytren’s disease (DD) has been acknowledged. However, the definitive causal relationship between these two factors and DD remains elusive. In order to establish a causal connection, we employed the two-sample Mendelian randomization method to evaluate the relationship between smoking and alcohol consumption and DD.

**Methods:**

Based on publicly available genome-wide association studies (GWAS), two-sample univariate MR analyses were performed to assess the causal effects of drinks per week, cigarettes per day, smoking initiation, age of initiation, and smoking cessation on DD. We used inverse variance weighted (IVW) to generate the primary results for the MR analysis. Furthermore, we performed sensitivity MR analyses based on various methods to assess the robustness of estimations. Bidirectional MR analyses were used to study the interaction between smoking and alcohol consumption. Multivariate MR analyses were used to obtain independent causal effects of smoking or drinking on DD.

**Results:**

Our two-sample MR, which was predominately based on IVW, revealed a causal relationship between drinks per week and DD (OR = 2.948, 95%CI: 1.746–4.975, *P* = 5.16E-05). In addition, there is no causal association between cigarettes per day, smoking initiation, age of initiation, smoking cessation and DD. Similar conclusions were reached by other MR methods. The results of the bidirectional MR analyses showed that the causal relationships between age of initiation and drinks per week were robust and significant. Multivariate MR results indicated that the causal effect of alcohol consumption on DD was independent of smoking.

**Conclusion:**

Our Mendelian Randomization study indicated that there is a causality between drinking alcohol and DD, but no such causality was found between smoking and DD. This is the first study to prove that drinking alcohol could cause DD. This could help people who are trying to prevent DD from happening in the first place.

**Supplementary Information:**

The online version contains supplementary material available at 10.1186/s12920-023-01650-4.

## Introduction

Dupuytren’s disease (DD), also referred to as palmar fascial fibromatosis, is a pathological condition characterized by the excessive growth of fibrous tissue in the palmar and finger regions. This leads to a progressive and irreversible flexion deformity in the metacarpophalangeal and interphalangeal joints, which can significantly impact hand function. Studies have indicated a high prevalence of DD in Nordic populations [1], with estimates of up to 8.2% globally [2]. Despite extensive research, the underlying cause of DD remains elusive. Smoking [3, 4] and alcohol consumption [5] have been recognized as common risk factors for numerous diseases. Nonetheless, certain lifestyle factors such as smoking and alcohol consumption have been proposed as potential risk factors forDD. Several studies have demonstrated a higher prevalence of DD in individuals with a history of smoking compared to the general population [6], and similarly, a correlation between higher lifetime cigarette consumption and average weekly alcohol consumption in DD patients compared to non-affected individuals [7, 8]. However, the causative relationship between these behaviors and DD remains a subject of ongoing debate and further research is required to establish a definitive conclusion.

Mendelian Randomization (MR) is a novelstatistical technique that utilizes single nucleotide polymorphisms (SNPs) as instrumental variables (IVs) for evaluating the causal pathway between putative risk factors and disease outcomes [9]. The random allocation of genetic variants during conception renders them less susceptible to confounding factors and reverse causality, making MR a valuable tool for investigating causal relationship. With the advent of genome-wide association studies (GWAS), the use of MR methods has gained prominence, enabling researchers to determine causal relationships between exposures and disease outcomes.

In this study, we performed MR analysis using SNPs that werestrongly associated with smoking and drinking behaviors to infer a causal relationship between them and the risk of DD.

## Method

### Study design

This study is atwo-sample MR analysis that utilized publicly available GWAS data and adhered to the three key assumptions of MR methodology: (i) a robust and significant correlation between the instrumental variables (IVs), which are SNPs, and smoking and drinking behavior; (ii) the IVs are uncorrelated with any confounding factors; and (iii) the IVs can only affect the risk of DD through their influence on smoking and drinking behavior. It is worth noting that all of the original studies included in this analysis were approved by the appropriate ethics review boards and obtained informed consent from the participants.

### Data sources

A summary of the genetic data pertaining to the phenotypes associated with smoking and drinking behavior was extracted from the most recent large-scale GWAS meta-analyses [10]. The studies involved a sample population of European individuals, with a sample size of over 3 million participants. The smoking phenotypes analyzed included age of initiation, smoking initiation, cigarettes per day, and smoking cessation, to evaluate the severity of smoking behavior. The regularity of drinking was assessed by evaluating the number of drinks consumed per week (drinks per week). All statistical tests were adjusted for demographic variables such as age, sex, and up to 20 genetic components. The European sample from the 1000 Genomes Project was utilized as a reference to eliminate any linkage disequilibria [11]. For this MR analysis, only SNPs with a genome-wide significance level (*P* < 5 × 10^–8^), a weak relationship with linkage disequilibrium (*r*^2^ < 0.001), and separated by more than 10,000 kb were extracted. Further information regarding the IVs used in the analysis can be found in Table 1.

**Table 1:** The source of SNPs for smoking and alcohol drink behaviour and for Dupuytren’s disease

Phenotype	Type	Sample size(N)	Consortium	Year	PMID	Source
Drinks per week	Continuous	941,280	GSCAN	2019	30,643,251	https://genome.psych.umn.edu/index.php/GSCAN
Cigarettes per day	Continuous	337,334	GSCAN	2019	30,643,251	https://genome.psych.umn.edu/index.php/GSCAN
Smoking initiation	Categorical	1,232,091	GSCAN	2019	30,643,251	https://genome.psych.umn.edu/index.php/GSCAN
Age of initiation	Continuous	341,427	GSCAN	2019	30,643,251	https://genome.psych.umn.edu/index.php/GSCAN
Smoking cessation	Categorical	547,219	GSCAN	2019	30,643,251	https://genome.psych.umn.edu/index.php/GSCAN
Dupuytren disease	Categorical	257,738	FinnGen	2022	-	https://r8.finngen.fi/

GSCAN GWAS and Sequencing Consortium of Alcohol and Nicotine use

The summary-level genetic data regarding DD was obtained from the Round 8 analysis conducted by the Finngen consortium (https://r8.finngen.fi/). The DD cases in the study were defined using the International Classification of Diseases, 10th Revision (ICD-10) code M72.0 or version 9 code 7286, and the sample size consisted of 257,738 individuals, including 4616 DD patients and 253,122 controls. The participants were all of European ancestry.

### Mendelian randomization analysis

The strength of the association between the genetic factors and the hypothesized risk factors was quantified using the F statistic. The power of each SNP was evaluated using the F statistic (F = Beta^2^/Se^2^) [12], and SNPs with an F statistic < 10 were considered weak instrumental variables and excluded from the analysis. To determine the causal relationship between smoking or drinking behavior and DD, the random-effect inverse variance weighted method (IVW) was used as the primary outcome. The IVW method is known to provide accurate estimates when there is absence of horizontal pleiotropic balance and weak instrumental bias [13]. The results were supplemented by MR-Egger, robust adjusted profile score (RAPS), weighted median (WME), and simple median (SME). The MR-Egger regression can correct for potential pleiotropic imbalance, however, it may lack power [14]. The RAPS method accounts for measurement errors in SNP-exposure effects and provides relatively unbiased estimates in the presence of multiple or weak instruments [15]. The median method (weighted median or simple median) is consistent when 50% of the genetic variation is ineffective as an instrumental variable [16]. The results of these methods can differ based on different assumptions in MR testing, and if they show consistent results, it suggests robust causality inference. The Mendelian Randomization Pleiotropy RESidual Sum and Outlier (MR-PRESSO) [17] method was also adopted, which involves a global test for horizontal pleiotropy, an outlier test to eliminate abnormal SNPs and estimate corrected results, and a distortion test to evaluate differences in pre- and post-correction results. If there are outliers, causality is re-evaluated after culling. We explored the causal associations of smoking and drinking behaviours with DD by using the above five MR methods. In order to investigate the interaction between smoking and alcohol consumption, we explored the bidirectional causal association between smoking and alcohol consumption based on univariate MR methods. In addition, considering that smoking and alcohol consumption may confound each other in the pathogenesis of DD, we used a multivariate MR approach to adjust for smoking and alcohol consumption separately to obtain an independent causal effect of smoking or alcohol consumption on the pathogenesis of DD [18], and assessed the strength of the genetic instrumental variables in a multivariate framework using conditional F-values [19]. In addition, we performed the following sensitivity analyses. The *P* value of the intercept in MR-Egger regression (< 0.05) was used as an indicator of horizontal pleiotropy. The Cochrane Q test was used to assess the heterogeneity of the causal effect estimates for each SNP on smoking and drinking behaviors. The leave-one-out analysis was conducted to determine whether a single SNP drives the causal estimation. To address the multiple testing issue, a Bonferroni-corrected *P* value of 0.01 (0.05/5) was applied to indicate statistical significance. All *P* values were two-tailed. The MR analyses were performed using the ‘TwoSampleMR’ package (version 0.5.6) [20], ‘MR-PRESSO’ package (version 1.0), ‘MendelianRandomization’ package (version 0.7.0) and ‘MVMR’ package (version 0.3) based on R software (version 4.2.1; The R Foundation for Statistical Computing, Vienna, Austria).

## Results

### Selection of instrumental variable

The study screened varying quantities of SNPs as instrumental variables for each exposure of interest, including drinking behavior and smoking behavior. The number of SNPs serving as instrumental variables for drinks per week, cigarettes per day, smoking initiation, age of initiation, and smoking cessation were 36, 21, 80, 6, and 6, respectively. All instrumental variables displayed an F statistic greater than 10, which signifies the absence of weak instrumental bias (refer to Additional file 1 and Tables S1-S5 for further information).

### The total causal effect between smoking or drinking behavior and DD

The results of the analysis suggested a causal association between genetically predicted drinks per week and an increased risk of DD. The odds ratio (OR) for DD obtained from the IVW was 2.948 (95% CI: 1.746–4.975, *P* = 5.16E-05). While the MR-Egger regression analysis did not yield a significant correlation (OR = 3.272, 95% CI: 0.886–12.077, *P* = 8.41E-02), the RAPS (OR = 2.962, 95% CI: 1.722–5.097, *P* = 8.73E-05), weighted median (OR = 2.847, 95% CI: 1.289–6.289, *P* = 1.03E-02), and simple median (OR = 3.007, 95% CI: 1.347–6.709, *P* = 6.86E-03) all supported significant causality (Table 2). The MR-PRESSO method did not detect any outliers and thus did not provide an estimate.

**Table 2:** MR results for the effect of smoking and alcohol drink behaviors on Dupuytren’s disease

Behaviour	SNPs, N	Method	OR	95%CI	*P* value	P for pleiotropy	P for Cochran’s Q	P for global test
Drinks per week	36	IVW	2.948	(1.746,4.975)	**5.16E-05**	0.864	0.428	0.429
		MR-Egger	3.272	(0.886,12.077)	8.41E-02			
		RAPS	2.962	(1.722,5.097)	**8.73E-05**			
		WME	2.847	(1.289,6.289)	**1.03E-02**			
		SME	3.007	(1.347,6.709)	**6.86E-03**			
		MR-PRESSO	-	-	-			
Cigarettes per day	21	IVW	1.051	(0.850,1.299)	6.45E-01	0.907	0.675	0.685
		MR-Egger	1.080	(0.650,1.793)	7.68E-01			
		RAPS	1.023	(0.821,1.276)	8.34E-01			
		WME	0.936	(0.686,1.277)	6.79E-01			
		SME	0.885	(0.635,1.231)	4.69E-01			
		MR-PRESSO	-	-	-			
Smoking initiation	80	IVW	1.162	(0.917,1.472)	2.12E-01	0.261	0.016	0.017
		MR-Egger	2.267	(0.695,7.399)	1.78E-01			
		RAPS	1.112	(0.873,1.416)	3.87E-01			
		WME	1.190	(0.870,1.628)	2.75E-01			
		SME	1.089	(0.806,1.471)	5.76E-01			
		MR-PRESSO	-	-	-			
Age of initiation	6	IVW	0.472	(0.131,1.702)	2.51E-01	0.492	0.117	0.147
		MR-Egger	2.027	(0.036,111.834)	7.47E-01			
		RAPS	0.354	(0.126,0.995)	4.91E-01			
		WME	0.401	(0.101,1.593)	1.94E-01			
		SME	0.275	(0.073,1.025)	5.40E-02			
		MR-PRESSO	-	-	-			
Smoking cessation	6	IVW	0.719	(0.462,1.119)	1.44E-01	0.739	0.552	0.630
		MR-Egger	0.962	(0.183,5.058)	9.66E-01			
		RAPS	0.715	(0.451,1.134)	1.54E-01			
		WME	0.749	(0.434,1.291)	2.98E-01			
		SME	0.766	(0.458,1.280)	3.09E-01			
		MR-PRESSO	-	-	-			

*Abbreviations: SNPs* Single nucleotide polymorphisms, *OR* odds ratio, *CI* Confidence interval, *IVW* Inverse variance weighted, *RAPS* Robust adjusted profile score, *WME* Weighted median, *SME* Simple median, *MR-PRESSO* Mendelian randomization pleiotropy residual sum and outlier

Regarding smoking behavior, there was no causal relationship with DD established. The results from the IVW method showed that the association between cigarettes per day (OR = 1.051, 95%CI 0.850–1.299, *P* = 6.45E-01), smoking initiation (OR = 1.162, 95%CI 0.917–1.472, *P* = 2.12E-01), age of initiation (OR = 0.472, 95%CI 0.131–1.702, *P* = 2.51E-01), smoking cessation (OR = 0.719, 95%CI 0.462–1.119, *P* = 1.44E-01) and DD lacked causality (Table 2). The results were further supported by sensitivity analyses using other MR methods, which also failed to establish a causal relationship. MR-PRESSO did not identify any outliers, and therefore no additional estimates were generated.

### The bidirectional causal correlation between smoking and drinking behavior

According to the IVW results, there was a positive causal association between drinks per week and smoking Initiation (OR = 1.376, 95% CI: 1.122–1.688, *P* = 2.21E-03) and vice versa (OR = 1.138, 95% CI: 1.099–1.178, *P* = 3.55E- 13), while age of initiation had a negative causal association with drinks per week (OR = 0.782, 95% CI: 0.677–0.902, *P* = 7.71E-04). However, after combining the two MR methods of sensitivity analysis (MR-Egger,weighted median), only the causal association from smoking Initiation to drinks per week was robust and significant (Table 3).

**Table 3:** MR results for the correlation between smoking and alcohol drink behaviors

Exposure	Outcome	SNP, N	Method	OR	95% CI	*P*-value
Drinks per week	Age of initiation	37	IVW	0.96	(0.866,1.064)	4.38E-01
			MR-Egger	1.073	(0.909,1.266)	4.12E-01
			WME	1.053	(0.970,1.142)	2.18E-01
Drinks per week	Cigarettes per day	37	IVW	1.063	(0.886,1.276)	5.12E-01
			MR-Egger	0.889	(0.661,1.198)	4.45E-01
			WME	0.995	(0.844,1.172)	9.48E-01
Drinks per week	Smoking cessation	36	IVW	1.066	(0.908,1.252)	4.34E-01
			MR-Egger	1.029	(0.779,1.360)	8.39E-01
			WME	1.066	(0.901,1.263)	4.55E-01
Drinks per week	Smoking initiation	36	**IVW**	**1.376**	**(1.122,1.688)**	**2.21E-03**
			MR-Egger	0.911	(0.678,1.224)	5.41E-01
			WME	0.96	(0.851,1.083)	5.06E-01
Age of initiation	Drinks per week	7	**IVW**	**0.782**	**(0.677,0.902)**	**7.71E-04**
			MR-Egger	1.199	(0.886,1.623)	2.92E-01
			WME	0.723	(0.637,0.820)	4.56E-07
Cigarettes per day	Drinks per week	22	IVW	1.013	(0.985,1.042)	3.73E-01
			MR-Egger	0.977	(0.932,1.024)	3.34E-01
			WME	0.997	(0.977,1.018)	8.12E-01
Smoking cessation	Drinks per week	8	IVW	0.987	(0.914,1.066)	7.44E-01
			MR-Egger	0.913	(0.715,1.165)	4.91E-01
			WME	0.951	(0.905,0.999)	4.50E-02
Smoking initiation	Drinks per week	85	**IVW**	**1.138**	**(1.099,1.178)**	**3.55E-13**
			MR-Egger	1.234	(1.036,1.471)	2.10E-02
			WME	1.129	(1.095,1.164)	1.04E-14

*Abbreviations: SNPs* Single nucleotide polymorphisms, *OR* OR odds ratio, *CI* Confidence interval, *IVW* Inverse variance weighted, *WME* Weighted median

### The direct causal effect between smoking or drinking behavior and DD

The direct causal effect of smoking and drinking behaviours on DD was obtained from the results of the multivariable MR-IVW approach. The causal association between alcohol consumption and DD was positive and significant (OR > 1, *P* < 5E-02) regardless of which smoking behaviours were adjusted for, and the conditional F-value was > 10, implying that the instrumental variables were valid. And when adjusted for alcohol consumption, there was no causal association between smoking and DD (*P* > 5E-02). The results of multivariate MR are available in Table 4.

**Table 4:** Multivariable MR results for smoking or alcohol drink behaviors on DD after adjusting for each other

Exposure	Conditional F-statistics	OR	95%CI	*P*-value
(Age of Initiation + Drinks Per Week)				
Age of Initiation	9	0.735	(0.316,1.712)	4.76E-01
DrinksPerWeek	65.3	3.042	(1.763,5.251)	6.47E-05
(Cigarettes Per Day + Drinks Per Week)				
Cigarettes Per Day	24.8	1.016	(0.827,1.247)	8.79E-01
Drinks Per Week	48.2	2.871	(1.725,4.778)	3.40E-05
(Smoking Cessation + Drinks Per Week)				
Smoking Cessation	9.2	0.768	(0.497,1.188)	2.36E-01
Drinks Per Week	38.9	2.919	(1.708,4.990)	8.93E-05
(Smoking Initiation + Drinks Per Week)				
Smoking Initiation	30.5	1.024	(0.803,1.305)	8.49E-01
Drinks Per Week	21.8	2.434	(1.361,4.355)	2.72E-03

*Abbreviations: OR* Odds ratio, *CI* Confidence interval

^a^ The results for multivariable Mendelian randomization analyses were based on multivariable inverse variance weighted method

### Sensitivity analysis

**Fig. 1:**
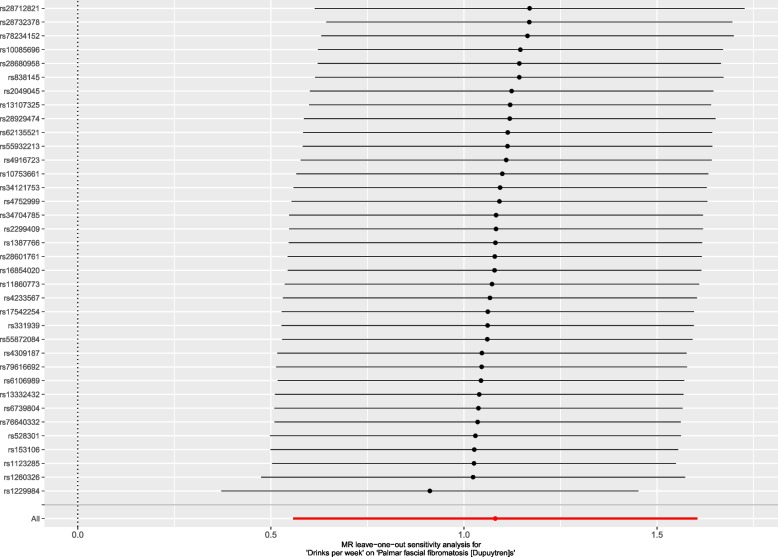
The leave-one-out analysis for drinks per week on DD

In relation to the genetically predicted drinks per week, the results of the MR-Egger intercept test and MR-PRESSO global test indicate the absence of horizontal pleiotropy, as the *P* values were not significant (*P* = 0.864 and *P* = 0.429, respectively) (Table 2). The Cochrane’s Q test also failed to detect heterogeneity (*P* = 0.428). The leave-one-out analysis found that no single SNP had a major impact on the relationship between drinks per week and DD risk, and the conclusion was deemed robust (Fig. 1).

Regarding smoking behavior, the MR-Egger intercept test and MR-PRESSO global test did not reveal significant bias for directional pleiotropy for the variables of cigarettes per day, age of initiation, and smoking cessation, as their *P* values were all greater than 0.05, indicating no heterogeneity. Only smoking initiation showed evidence of pleiotropy (*P* = 0.017 < 0.05) and heterogeneity (*P* = 0.016 < 0.05) based on the results of the global test and Cochran’s Q test, respectively (Table 2). However, results from various MR methods still showed a consistent pattern.

## Discussion

This study represented a novel examination using the two-sample MR approach to investigate the potential causal link between smoking and drinking behavior and DD in European populations. The results indicated a significant causal association between alcohol consumption and an increased risk of DD, while there is insufficient evidence to support a causal connection between smoking behavior and DD.

The findings of the present study indicated a causal relationship between increased frequency of alcohol consumption and a heightened risk of DD in European populations. This conclusion aligns with the results of some previous studies, including a large cohort study in France which found a significant positive association between heavy drinking and the incidence of DD (OR = 1.36, 95% CI 1.10–1.69) [21], and in miner populations where a significantly increased risk of DD was observed in alcoholics (OR = 1.59, 95% CI 1.47–1.72) [22]. The evidence suggested a dose-dependent relationship between alcohol intake and DD risk [8]. However, not all studies have reached this conclusion. A nested case–control study in the Icelandic population found no difference in self-reported alcohol consumption between men with and without DD [23]. This discrepancy may be due to the use of self-reported lifetime drinking levels (little, moderate, heavy) rather than quantified alcohol consumption in the assessment of drinking status, which may have masked the link between drinking and DD. Our study, based on the latest data from large-scale GWASs, confirmed alcohol consumption as a risk factor for DD.

Several proposed mechanisms have been put forth to explain the link between alcohol intake and the risk of developing DD, although limited research has been conducted in this area. One potential explanation is that alcohol consumption can result in the increased production of reactive oxygen species (ROS) within the body, which can cause oxidative stress, resulting in cellular and tissue damage [24, 25]. Oxidative stress, in turn, has been linked to several chronic diseases and conditions [26]. Another mechanism is that alcohol consumption has been shown to increase the production of growth factors, such as transforming growth factor-beta (TGF-β), which play a role in the formation of fibrous tissue [27]. This increase in growth factors can lead to the proliferation and differentiation of fibroblasts, contributing to an increase in fibrous tissue in the hand [28]. Additionally, alcohol consumption can cause inflammation in various tissues, including the hand, which can increase the production of growth factors and enzymes involved in the formation of fibrous tissue, as well as exacerbate oxidative stress, further contributing to the development of DD [29, 30]. Lastly, alcohol consumption can interfere with the metabolism of collagen, a protein essential for the strength and structure of connective tissues, by reducing its synthesis by fibroblasts and increasing the production of collagen-degrading enzymes. This can lead to a decreased ability of the tissue to resist stress and strain, thereby increasing the likelihood of developing DD [31, 32].

The relationship between smoking and the risk of developing DD is a matter of debate, with varying results from different studies. A study by An et al. determined a statistical association between smoking and DD through case comparison [6]. Another case–control study by Burge et al. [7] of 222 patients with DD found a strong association between current smoking and DD requiring surgery (OR = 2.8, 95% CI 1.5–5.2). Burke et al. [22] conducted a continuous sample of 97,537 miners and determined that the risk of DD was significantly elevated in heavy smokers (OR = 1.31, 95% CI 1.17–1.47). These studies all utilized cross-sectional designs. However, Descatha et al. [21], after adjusting for variables based on a large cohort with nearly 20 years of data taking into account participants’ smoking status, found that the degree of smoking was not significantly linked to the incidence of DD. Despite attempting MR analysis to investigate the potential association between smoking behaviors and DD, no causal association was found.

The current study offered several advantages over previous research. Firstly, it provided a comprehensive evaluation of the causal relationship between smoking and alcohol consumption and the risk of DD, contributing to a deeper understanding of the risk factors associated with DD and serving as a foundation for its prevention. Secondly, the study quantified cigarette and alcohol consumption more accurately by recording the amount consumed instead of self-reported heaviness thus could achieve a more reliable estimate. Thirdly, the study utilized a large cohort sample size in the GWAS dataset, making the effect values more robust and powerful compared to smaller studies based on individual-level data. Fourthly, the MR analysis was performed while ensuring that underlying assumptions were met, reducing the likelihood of bias due to confounding variables or reverse causation. Fifthly, we delved deeper into the interaction between smoking and drinking behaviors using bidirectional MR analyses. In addition, we conducted multivariable MR analyses to disentangle the direct causal impacts of smoking and drinking behaviors on DD. As a result, the findings of this study offered reliable evidence supporting a causal relationship between alcohol consumption and DD, and demonstrated that smoking is indeed a risk factor for DD. Finally, the study population was limited to individuals of European descent, which minimizes the potential impact of population stratification on the results.

However, this study also had some limitations that should be acknowledged. Firstly, we were unable to examine the effect of gender due to the unavailability of demographic data from the participants in the GWAS dataset. Secondly, the relationship between alcohol consumption and DD could potentially be nonlinear, however, our MR analysis was limited to summary data level, so we were unable to explore this further. Thirdly, as there are currently no known definitive causally-related risk factors for DD, our study was unable to perform multivariate and mediational analyses to determine a more specific causal pathway between alcohol consumption and DD. Finally, it is important to note that our study was limited to European populations, which may impact the generalizability of our findings to other populations.

## Conclusion

Our Mendelian Randomization study establishes a causal association between alcohol consumption and DD, unlike smoking, underscoring alcohol’s role in DD etiology. This pioneering finding may hold significance for targeted primary prevention strategies addressing DD prevalence.

### Supplementary Information

**Additional file 1.**

## Data Availability

The GWAS datasets for smoking and drinking behaviors analysed in this study are available in the website: https://genome.psych.umn.edu/index.php/GSCAN, and the GWAS dataset for Dupuytren’s disease is ID: M13_DUPUTRYEN from the website: https://r8.finngen.fi/:, respectively. The genetic data applied for this analysis can be downloaded with https://doi.org/10.6084/m9.figshare.23733621 and the R code to reproduce the results is available on GitHub: https://github.com/Wang97Zifeng/ArticleOne/.
