# Supplementary material for: Smoking, alcohol consumption and risk of Dupuytren’s disease: a Mendelian randomization study
Source: BMC Med Genomics. 2023 Sep 7;16:212. doi: 10.1186/s12920-023-01650-4 (PMC10483747; doi:10.1186/s12920-023-01650-4)

Supplementary Figure 1. Scatter plot for age of initiation.


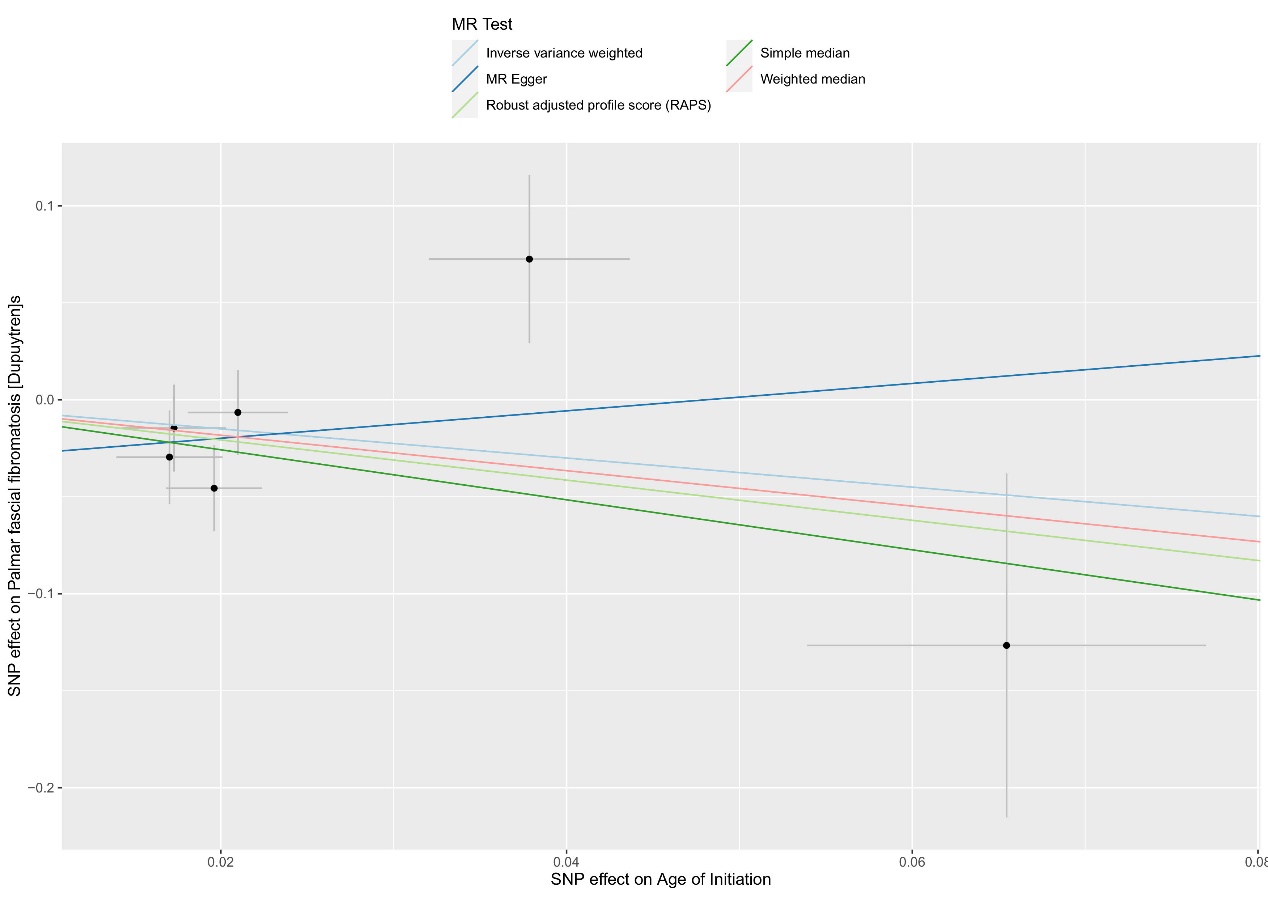


Supplementary Figure 2. Scatter plot for cigarettes per day.


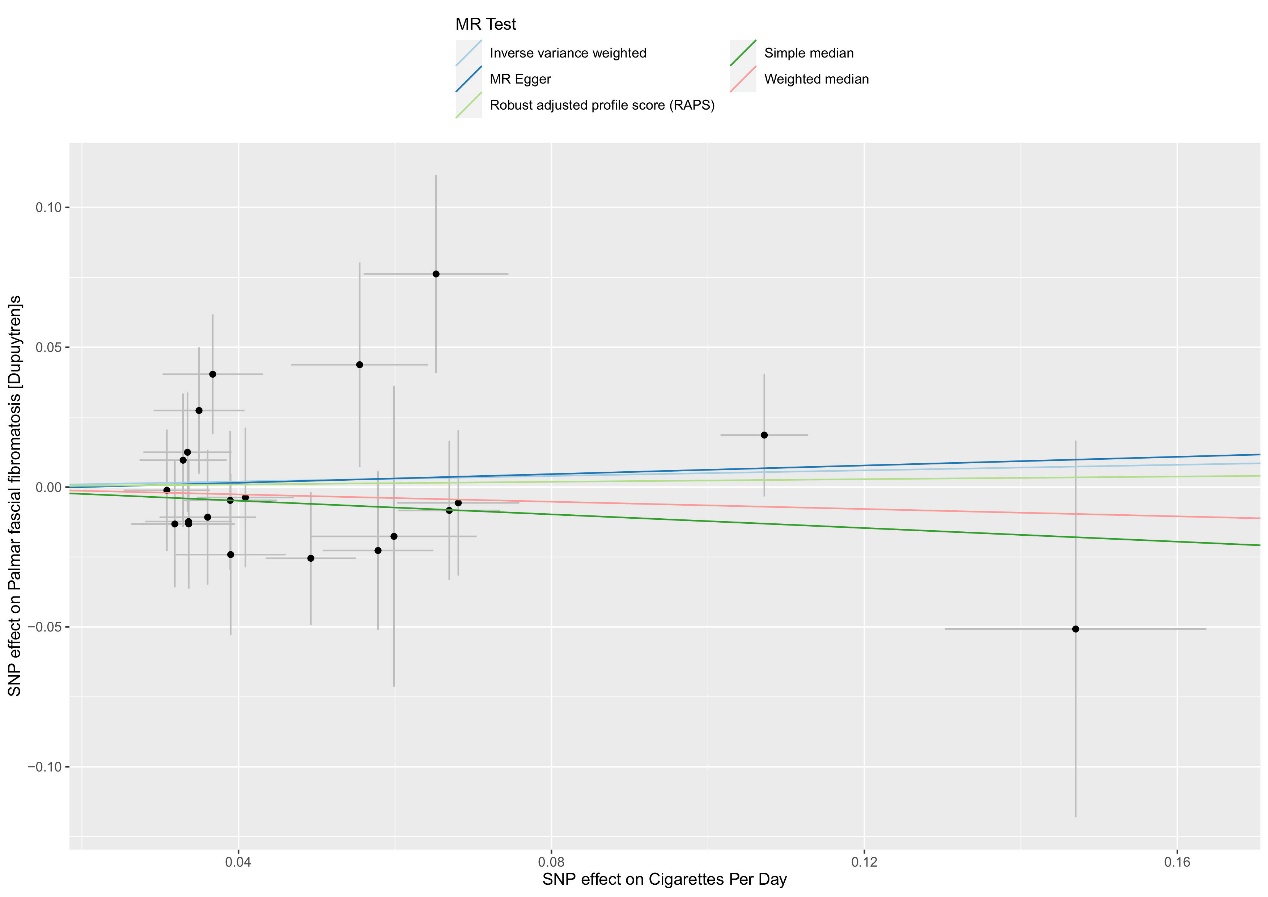


Supplementary Figure 3. Scatter plot for drinks per week.


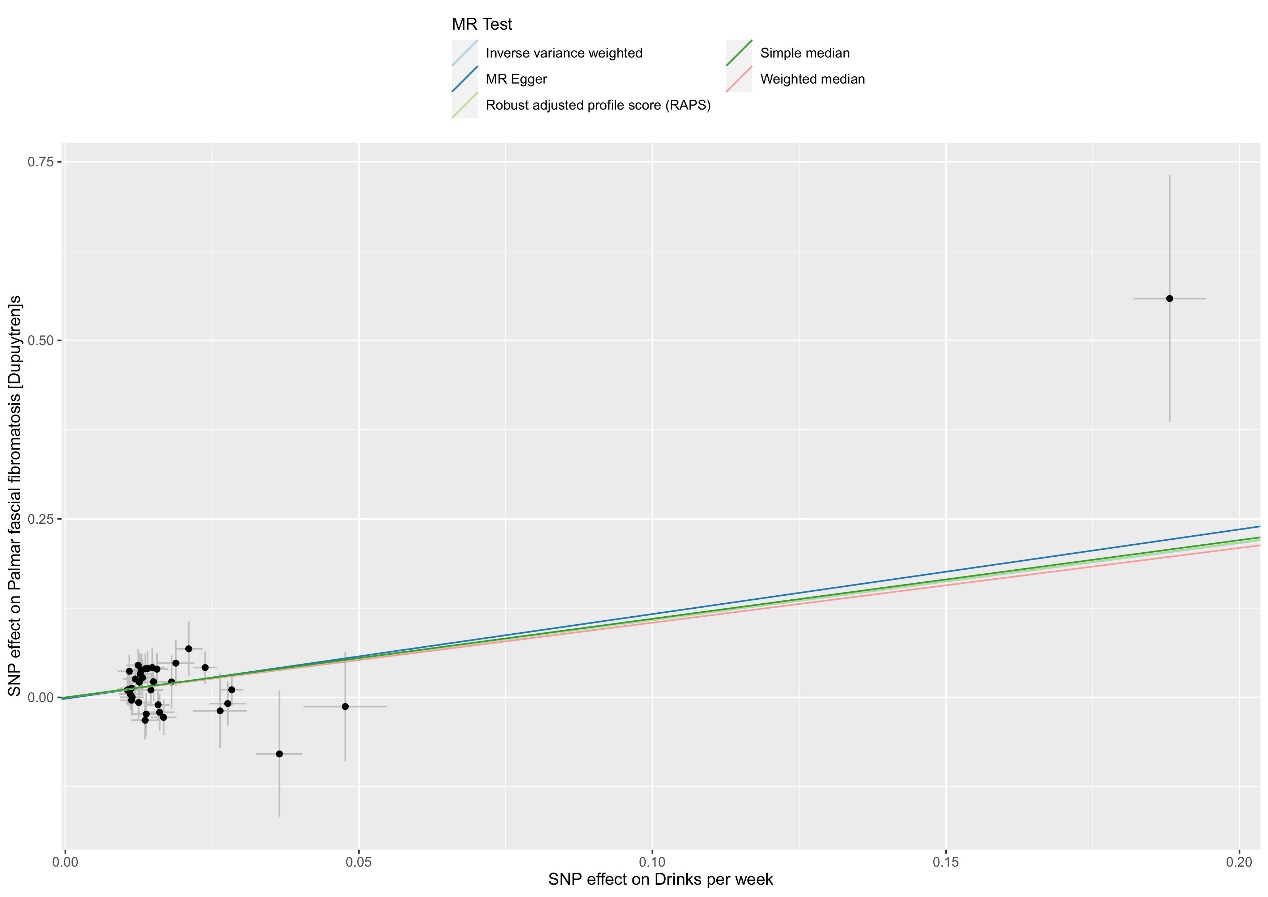


Supplementary Figure 4. Scatter plot for smoking cessation.


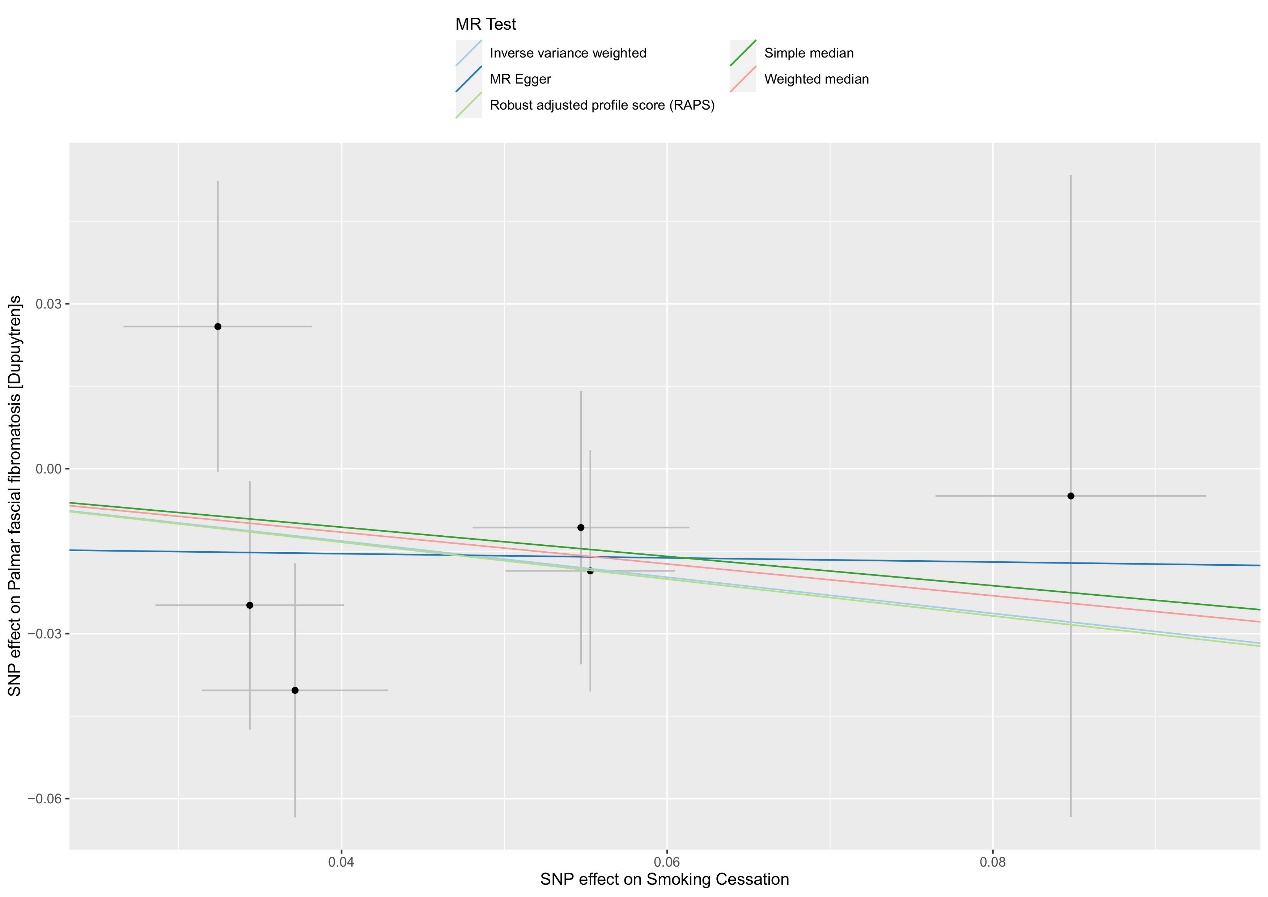


Supplementary Figure 5. Scatter plot for smoking initiation.


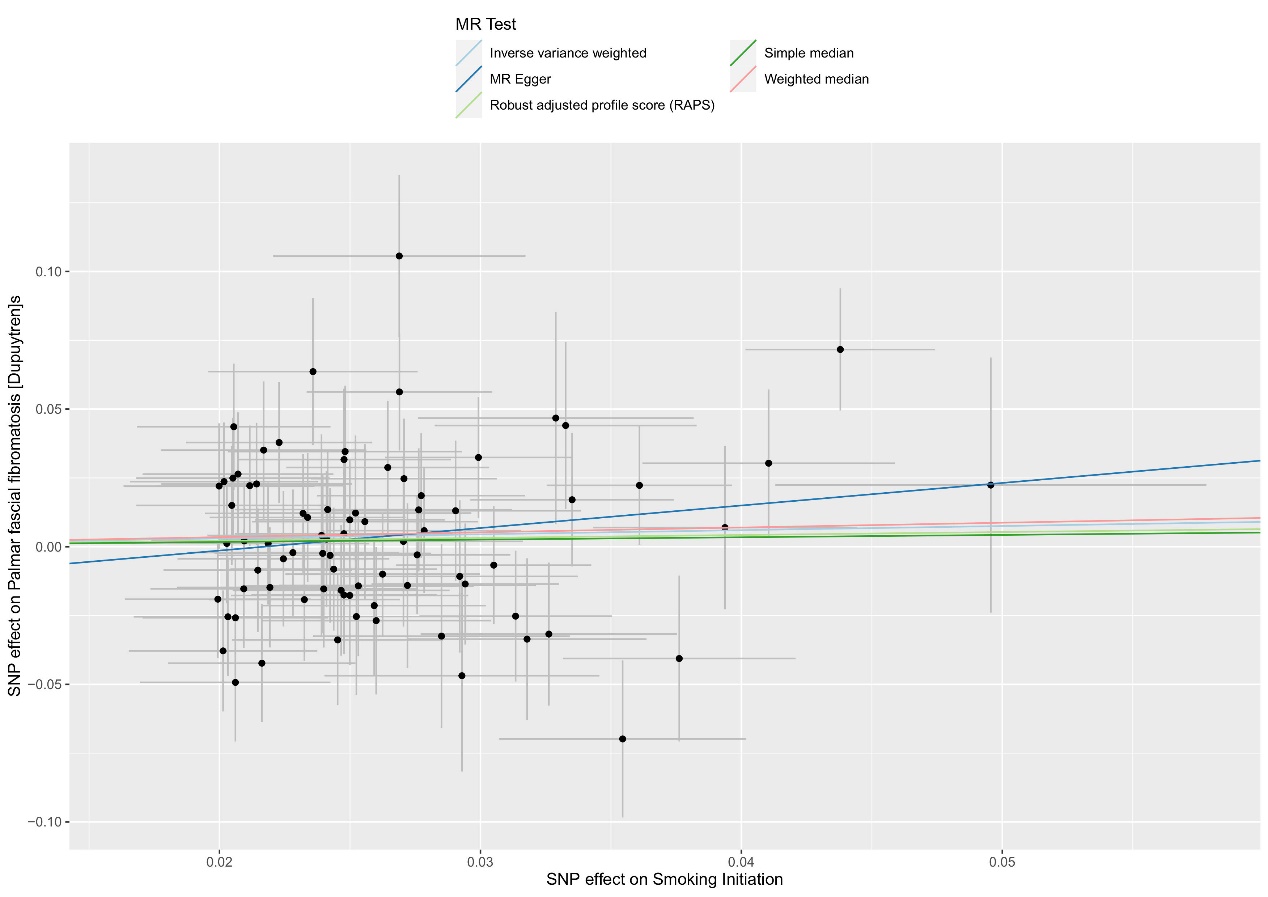

Supplement: Supplementary file 1 — Additional file 1. [file 12920_2023_1650_MOESM1_ESM.zip › Supplementary Tables and Figures/Supplementary Figures 1.docx]
