# Supplementary material for: Smoking, alcohol consumption and risk of Dupuytren’s disease: a Mendelian randomization study
Source: BMC Med Genomics. 2023 Sep 7;16:212. doi: 10.1186/s12920-023-01650-4 (PMC10483747; doi:10.1186/s12920-023-01650-4)

Supplementary Figure 6. Leave-one-out analysis for age of initiation.


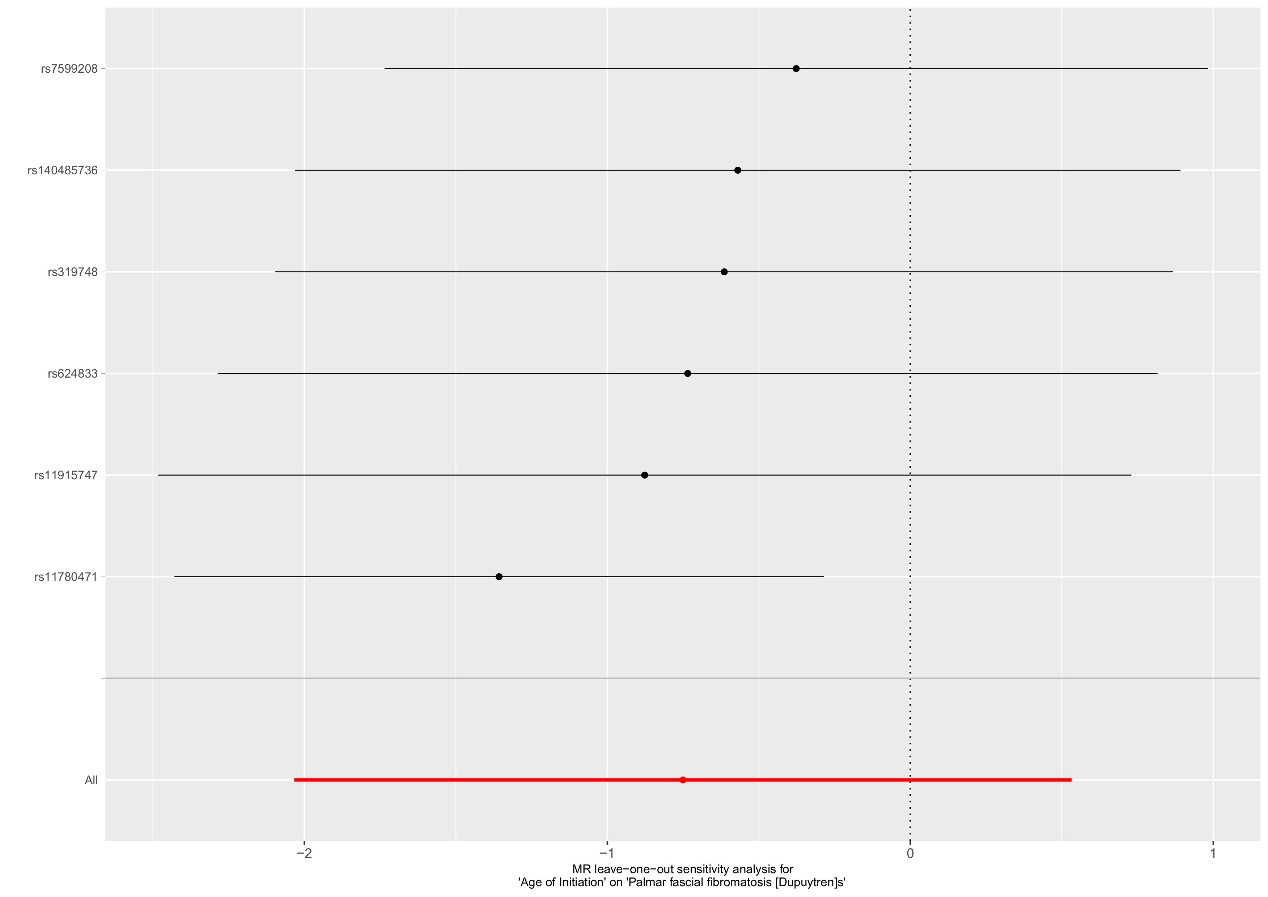


Supplementary Figure 7. Leave-one-out analysis for cigarettes per day.


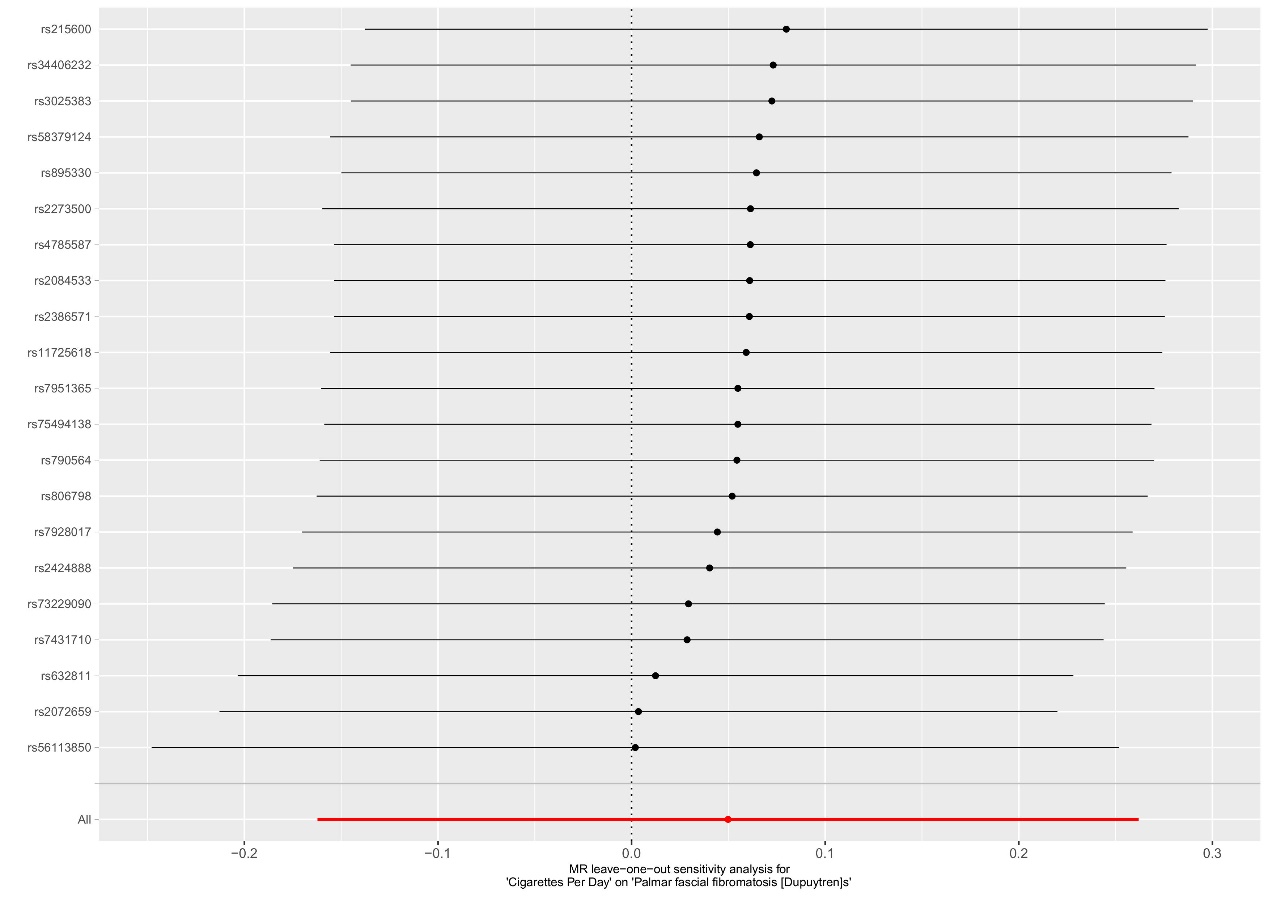


Supplementary Figure 8. Leave-one-out analysis for smoking cessation.


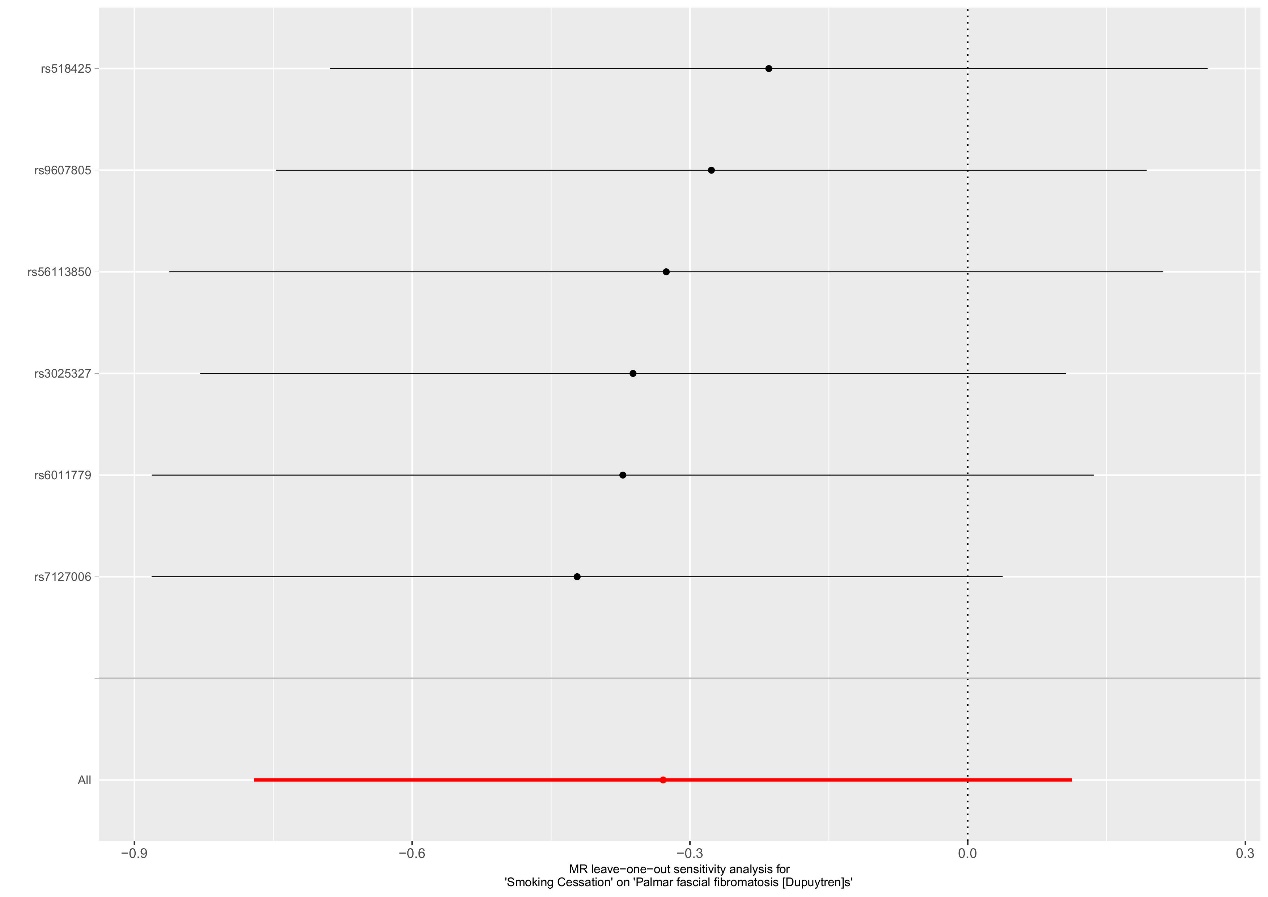


Supplementary Figure 9. Leave-one-out analysis for smoking initation.


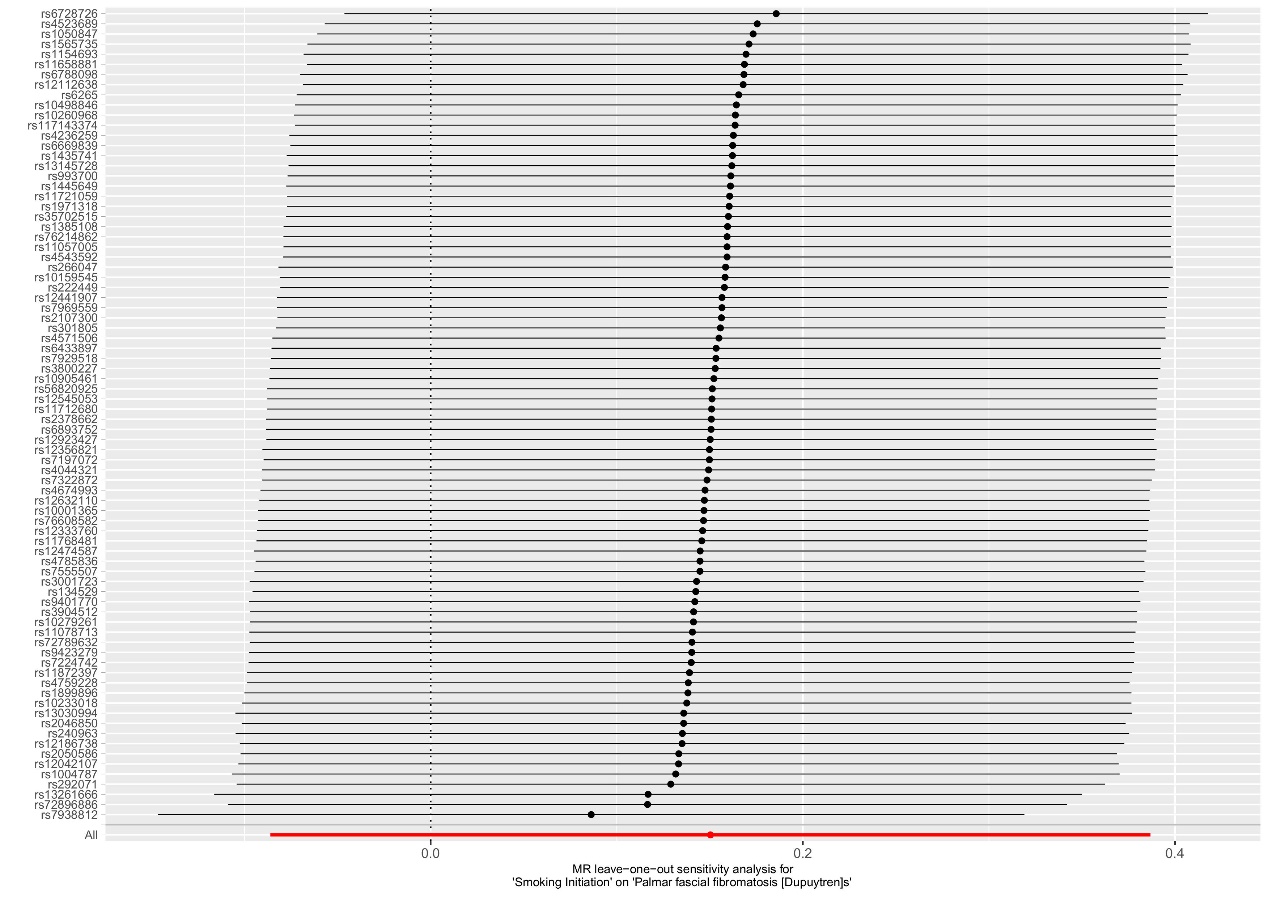

Supplement: Supplementary file 1 — Additional file 1. [file 12920_2023_1650_MOESM1_ESM.zip › Supplementary Tables and Figures/Supplementary Figures 2.docx]
