# Supplementary material for: Smoking, alcohol consumption and risk of Dupuytren’s disease: a Mendelian randomization study
Source: BMC Med Genomics. 2023 Sep 7;16:212. doi: 10.1186/s12920-023-01650-4 (PMC10483747; doi:10.1186/s12920-023-01650-4)

Supplementary Figure 7. Funnel plot for age of initiation.


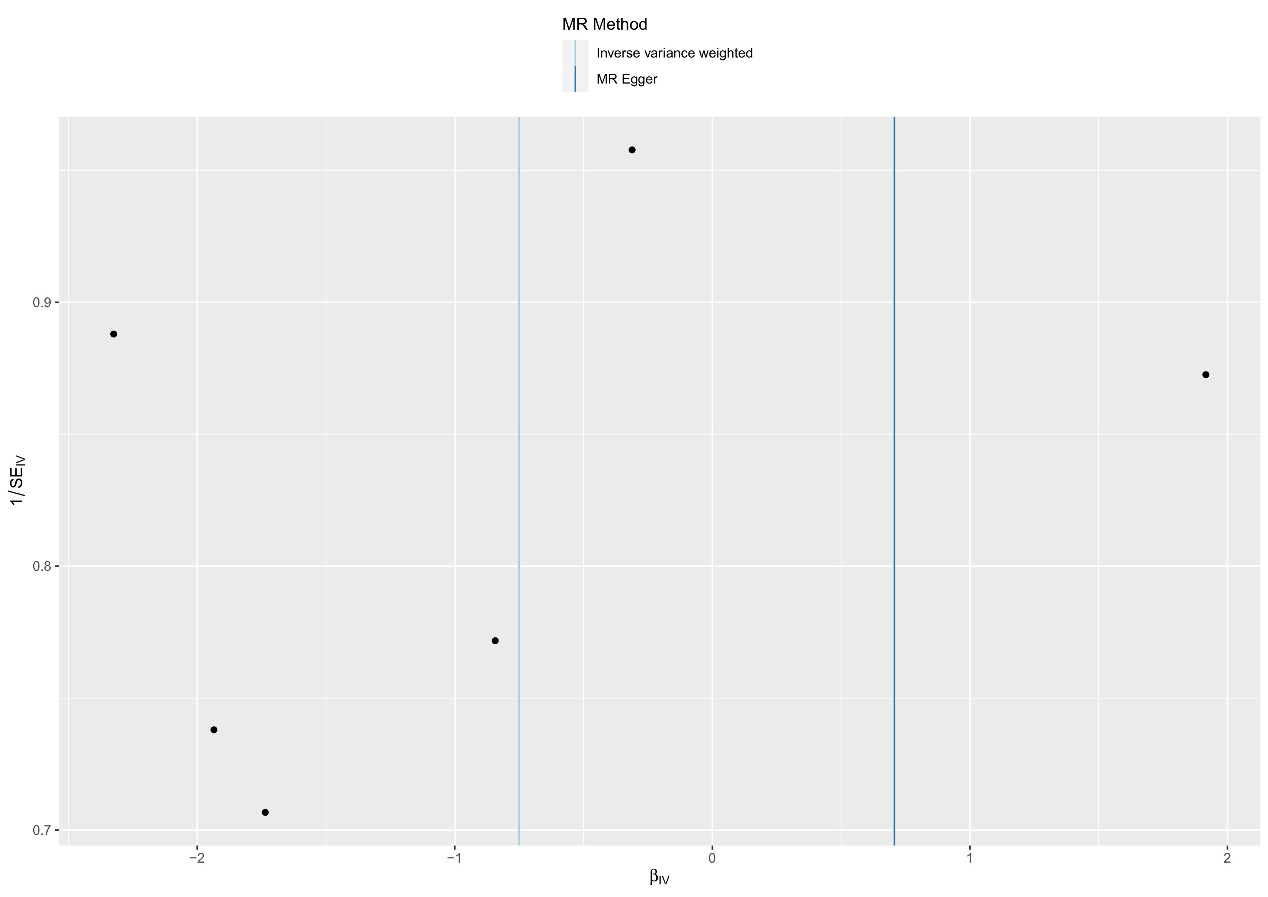


Supplementary Figure 8. Funnel plot for cigarettes per day.


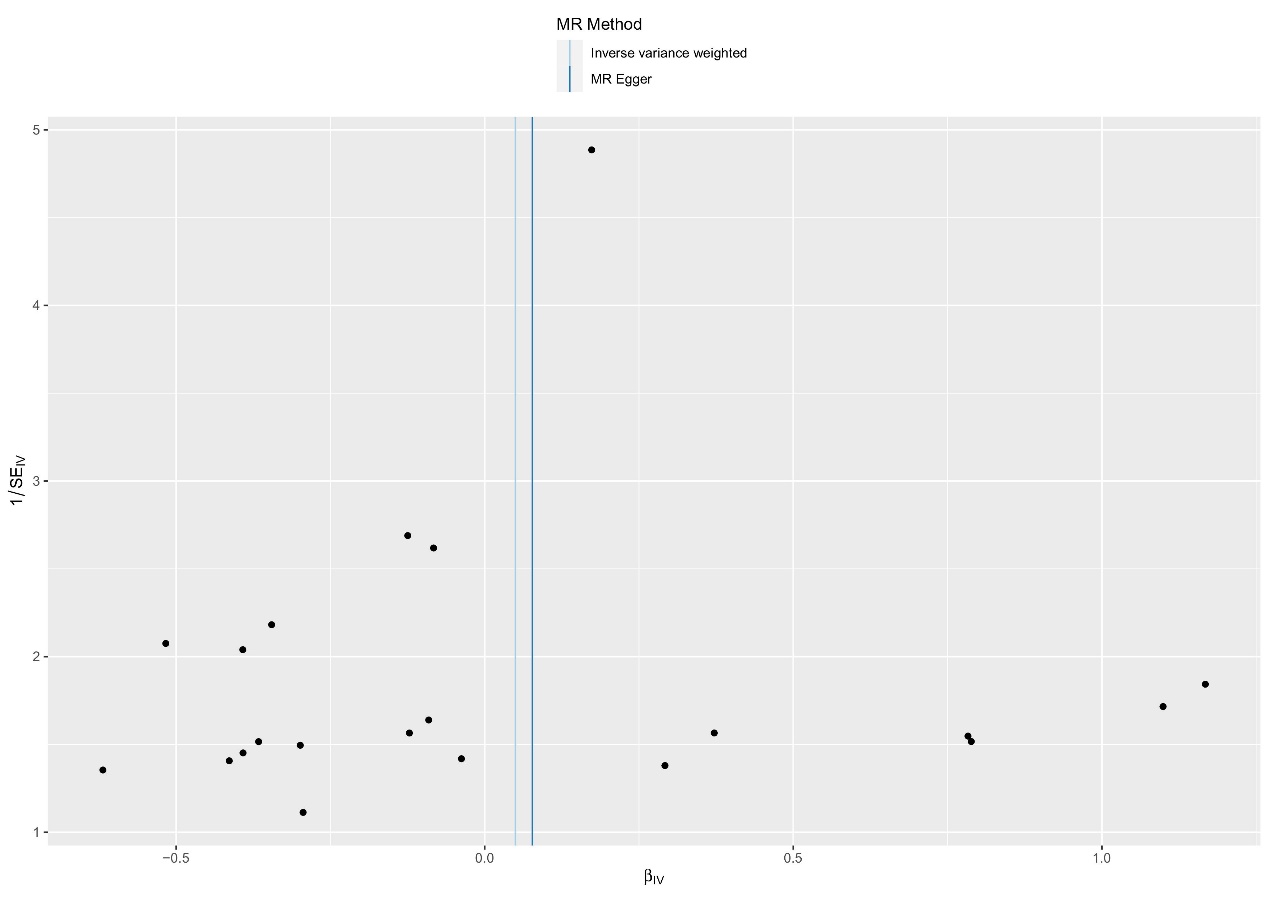


Supplementary Figure 9. Funnel plot for drinks per week.


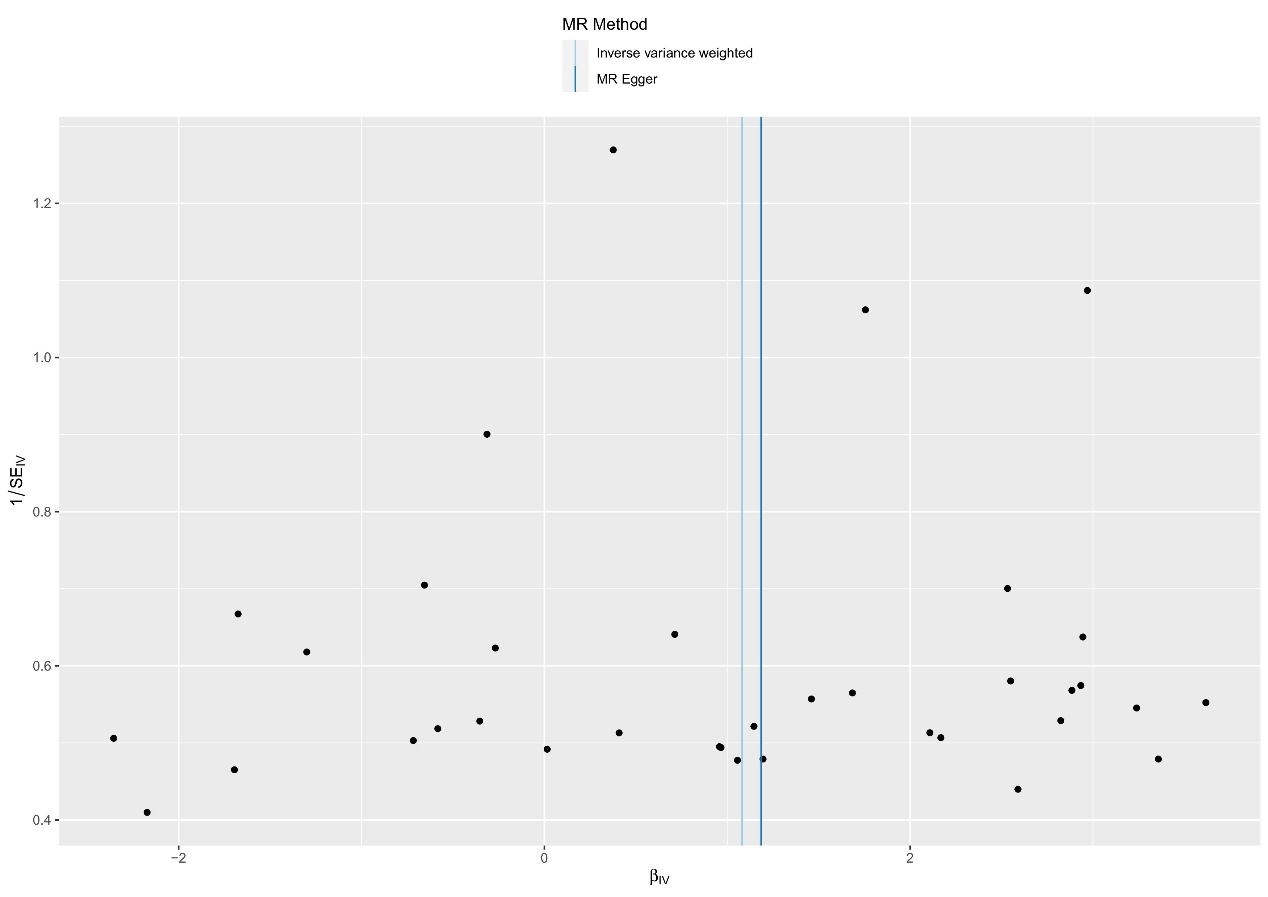


Supplementary Figure 10. Funnel plot for smoking cessation.


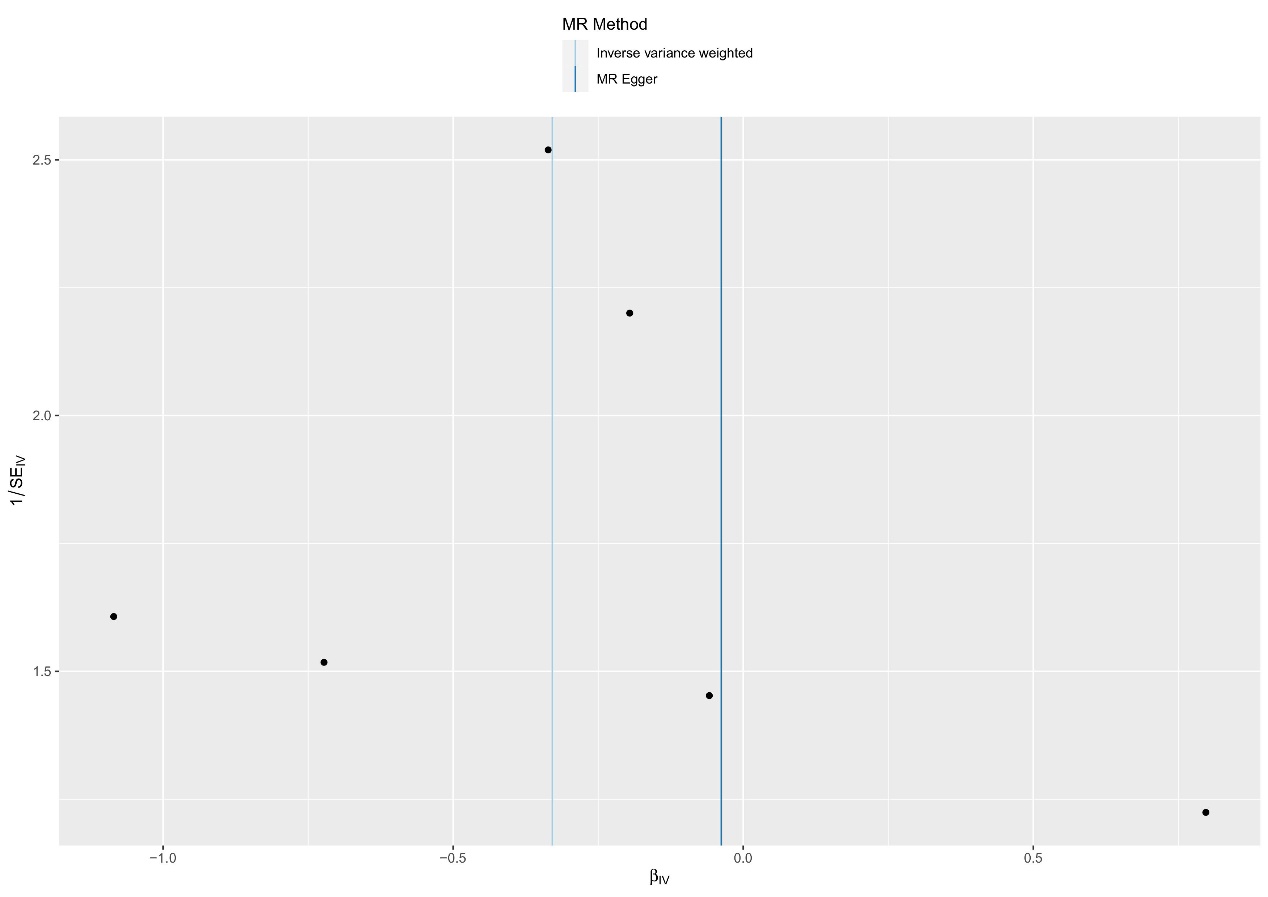


Supplementary Figure 11. Funnel plot for smoking initation.


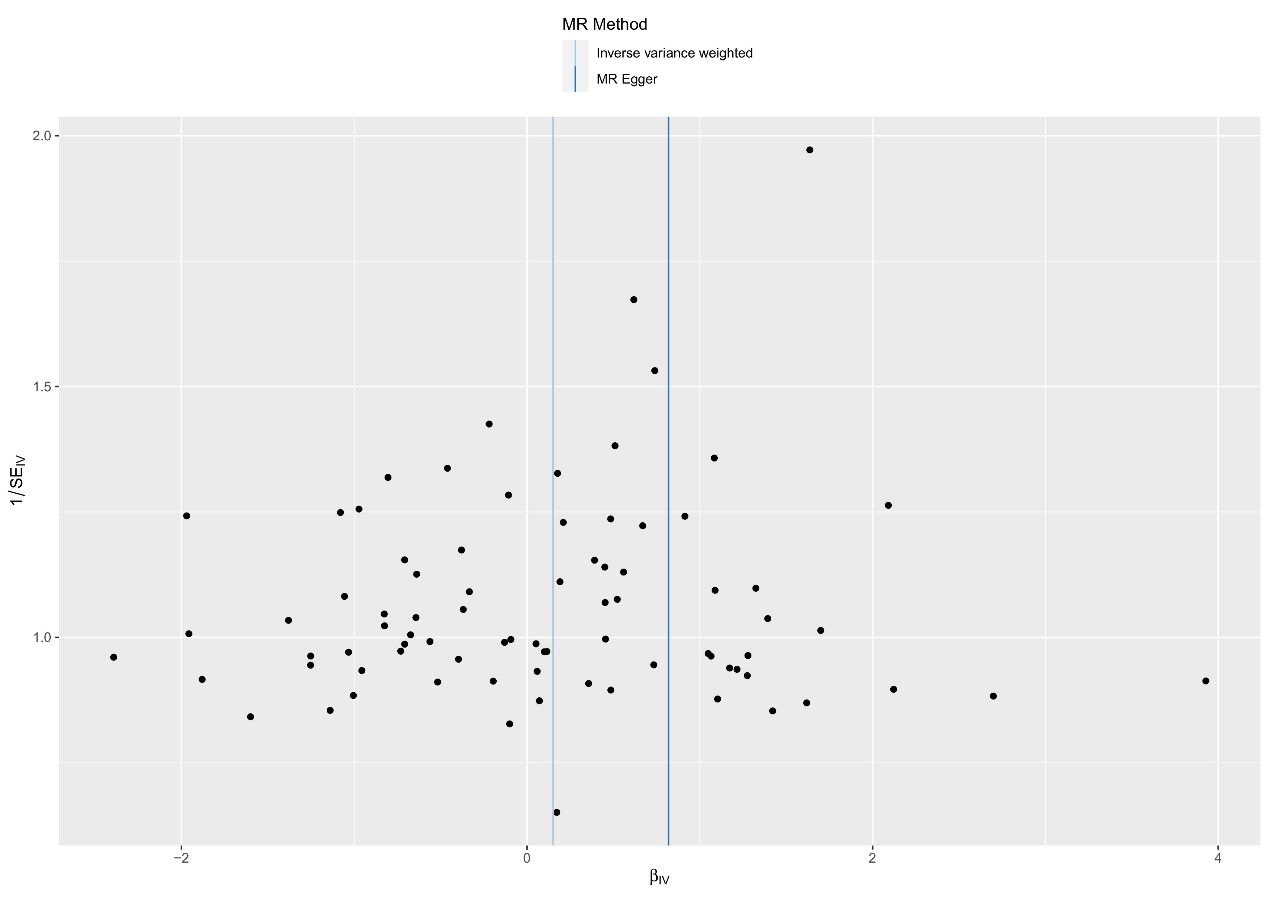

Supplement: Supplementary file 1 — Additional file 1. [file 12920_2023_1650_MOESM1_ESM.zip › Supplementary Tables and Figures/Supplementary Figures 3.docx]
